# Wheat Bran Pretreatment by Room Temperature Ionic Liquid-Water Mixture: Optimization of Process Conditions by PLS-Surface Response Design

**DOI:** 10.3389/fchem.2019.00585

**Published:** 2019-08-23

**Authors:** Monica Araya-Farias, Eric Husson, Jorge Saavedra-Torrico, Doriane Gérard, Romain Roulard, Isabelle Gosselin, Harivoni Rakotoarivonina, Virginie Lambertyn, Caroline Rémond, Catherine Sarazin

**Affiliations:** ^1^Unité de Génie Enzymatique et Cellulaire, UMR 7025 CNRS, Université de Picardie Jules Verne, Amiens, France; ^2^Escuela de Ingenieria de Alimentos, Pontificia Universidad Catolica de Valparaíso, Valparaíso, Chile; ^3^Chaire AFERE, UMR Fractionnement des AgroRessources et Environnement 614 INRA, Université de Reims Champagne-Ardenne, Reims, France; ^4^Plate-forme de Microscopie Electronique, Université de Picardie Jules Verne, Amiens, France

**Keywords:** wheat bran, room temperature ionic liquid, pretreatment, ionic liquid-water mixture, hemicellulolytic cocktail, enzymatic hydrolysis, partial least square surface response design

## Abstract

Room Temperature Ionic Liquids (RTILs) pretreatment are well-recognized to improve the enzymatic production of platform molecules such as sugar monomers from lignocellulosic biomass (LCB). The conditions for implementing this key step requires henceforth optimization to reach a satisfactory compromise between energy saving, required RTIL amount and hydrolysis yields. Wheat bran (WB) and destarched wheat bran (DWB), which constitute relevant sugar-rich feedstocks were selected for this present study. Pretreatments of these two distinct biomasses with various 1-ethyl-3-methylimidazolium acetate ([C2mim][OAc])-water mixtures prior to hydrolysis catalyzed by hemicellulolytic cocktail (Cellic CTec2) were finely investigated. The main operating conditions such as pretreatment temperature (25–150°C), time (40–180 min), WB and DWB loading (2–5% w/v) and concentration of [C2mim][OAc] in water [10–100% (v/v)] were screened through glucose and xylose yields and then optimized through a Partial Least Square (PLS)—Second Order Design. In an innovative way, the PLS results showed that the four factors and their interactions could be well-fitted by a second-order model (*p* < 0.05). The quadratic PLS models were used to predict optimal pretreatment conditions. Thus, maximum glucose (83%) and xylose (95%) yields were obtained from enzymatic hydrolysis of WB pretreated at 150°C for 40 min with 10% of [C2mim][OAc] in water and 5% of WB loading. For DWB, maximum glucose (100%) and xylose (57%) yields were achieved for pretreatment temperatures of 150°C and 25°C, respectively. The required duration was still 40 min, with 20% of [C2mim][OAc] in water and a 5% DWB loading. Then, Multiple Response Optimization (MRO) performed by Nelder-Mead Simplex Method displayed sugar yields similar to those obtained by individual PLS optimization. This complete statistical study confirmed that the established models were appropriate to predict the sugar yields achieved after different pretreatment conditions from WB and DWB biomasses. Finally, Scanning Electron microscopy (SEM) studies allowed us to establish clearer link between structural changes induced by pretreatment and the best enzymatic performances obtained.

## Introduction

Among the sustainable energy resources, lignocellulosic biomass (LCB) constitutes a vast and biorenewable source for producing high value-added molecules and fuels. LCB is composed of two main carbohydrate polymers (cellulose and hemicellulose) and an aromatic polymer (lignin) (Balat, 2011). Cellulose and hemicellulose are starting raw materials for the production of sugar which can be fermented for example into bioethanol while lignin is a potential source for the production of bioplastics, dispersants, and nanocomposites (Norgren and Edlund, 2014). However, as a complex matrix, LCB needs to be pretreated prior to conversion into chemicals and biofuels (Kumar et al., 2009; Alvira et al., 2010; Akhtar et al., 2016). Diluted acid hydrolysis, organosolv pulping, steam explosion have been proposed in the literature as effective pretreatments to improve the biomass digestibility by reducing its recalcitrance (Chandra et al., 2007; Hendriks and Zeeman, 2009; Alvira et al., 2010; Perez-Cantu et al., 2013; Xu et al., 2013; Shi et al., 2014). However, they exhibit some drawbacks such degrading fermentable sugars and producing by-products inhibitory toward biocatalysts (Hendriks and Zeeman, 2009; Ahmed et al., 2016; Akhtar et al., 2016; Elgharbawy et al., 2016). Hot water extraction pretreatment has been proposed as an environmental-friendly as water is the only agent used. For example, from woody biomass, hot water extraction in a pilot-scale allowed to achieve high release of glucose and xylose mainly originated from hemicellulose and also xylose oligomers. Reducing extraction time helped to limit sugars degradation although decreasing sugar yields (Yan and Liu, 2015; Yan et al., 2016).

Alternatively, some Room Temperature Ionic Liquids (RTILs) have achieved success as green solvents for biomass pretreatment due to their double ionic and organic nature that gives them advantageous properties including the ability to dissolve biopolymers, negligible vapor pressure, low flammability, chemical, and thermal stability (Li et al., 2009; Olivier-Bourbigou et al., 2010; Liu et al., 2012; Wang et al., 2012; Elgharbawy et al., 2016; Shi and Wang, 2016; Zhang et al., 2017). It has also been reported that some imidazolium-based RTILs with alkyl chain length of cation inferior to 4 carbons exhibit lower toxicity than those with alkyl chain superior to 4 carbons (Egorova and Ananikov, 2014). Up to date, most reported biomass pretreatment involves 1-ethyl-3-methylimidazolium acetate ([C2mim][OAc]) which have proved to be highly effective to breakdown the lignocellulosic complex and improve enzymatic hydrolysis (Sun et al., 2009; Fu and Mazza, 2011a; Shill et al., 2011; Auxenfans et al., 2012, 2014a, 2017b; Liu et al., 2012, 2016; Yoon et al., 2012; Brandt et al., 2013; da Costa Lopes et al., 2013; Liu and Ng, 2016; Papa et al., 2017; Perez-Pimienta et al., 2017). Moreover, imidazolium-based RTILs can be reused without loss of pretreatment efficiency (Shill et al., 2011; Auxenfans et al., 2012, 2014a,b) present biocompatibility with cellulolytic enzymes (Engel et al., 2012; Auxenfans et al., 2017b) and fermentative microorganisms (Mehmood et al., 2015; Ryu et al., 2015). Moreover, the feasibility of scale-up with this ionic liquid (IL) was demonstrated on switchgrass pointing out as well challenges still existing prior to pilot scale demonstration such as IL recycling and reuse, development of IL-tolerant enzyme cocktails or one-pot process for an economically viable process (Liang et al., in press).

One strategy involves minimizing of the required amount of RTIL in an integrated process. As water is already used in the process (regeneration step, enzymatic hydrolysis, and fermentation), mixing RTIL and water can reduce viscosity of medium facilitating the handling and recycling operations. Indeed, some studies have reported that the addition of water to RTIL can reduce the viscosity of medium and maintain the effectiveness of pretreatment even in the presence of high contents of water (Kamiya et al., 2008; Fu and Mazza, 2011a; Hou et al., 2013; Shi et al., 2013, 2014; Viell et al., 2016; Perez-Pimienta et al., 2017; Hu et al., 2018). On the contrary, several authors have reported that the pretreatment efficiency is drastically reduced when RTIL is mixed with water reinforcing the importance of the use of pure RTIL (Swatloski et al., 2002; Liu et al., 2008; Mazza et al., 2009; Doherty et al., 2010). This raised a dependency on the nature of the IL and the conditions of implementation. It is thus difficult to perform a fair conclusion about the real impact of RTIL-water mixture as biomass pretreatment which raises immediately a question: “Could we minimize the amount of RTIL without incurring a loss of pretreatment efficiency?” Recently, it was highlighted that the success of any pretreatment depends on the physico-chemical properties of the pretreated material as well as the conditions employed which can interact with each other in numerous complex ways (Badgujar and Bhanage, 2015; Papa et al., 2017). Consequently, optimization of pretreatment conditions is highly necessary not only to improve the performance of enzymatic hydrolysis but also to provide additional comprehensive information about the efficiency of the pretreatment. Optimization by the conventional “one-factor-at-one-time” (OFAT) method is time consuming and the interactions between the factors are not estimable from OFAT experiments (Czitrom, 1999). Experimental Design (ED) is an effective statistical approach to investigate and to optimize multivariate processes. To date, many types of LCB have been pretreated by RTILs and optimized by Response Surface Methodology (RSM) (Bajaj and Wani, 2011; Fu and Mazza, 2011b; Tan et al., 2011; Yoon et al., 2012; Lee et al., 2013; Sidik et al., 2013; Qiu et al., 2014; Singh et al., 2015; Wang et al., 2015; Li et al., 2017; Saha et al., 2017; Oliveira Ribeiro et al., 2018; Trinh et al., 2018). Only very few research groups have investigated the optimum conditions for RTIL-based pretreatment using another statistical design (Elgharbawy et al., 2017; Papa et al., 2017; Vergara et al., 2018). The standard Multiple Linear Regression (MLR), which is extensively described in the literature (Myers et al., 2016), requires that each one of variables in the X matrix must be independent (no collinearity and no auto-correlation) and fit to a Normal Statistical Distribution (Gauss). However, these conditions are not always met experimentally. Alternatively, Partial Least Square Regression (PLS) which is supported by the Nonlinear Iterative Partial Least Squares (NIPALS) algorithm (Wold et al., 2001) allows to analysis of a large number of variables, highly correlated and ill-conditioned matrices (Ferrer, 2007) which represents the main advantage of this method compared to MLR.

In the present study, wheat bran (WB) and destarched wheat bran (DWB) were selected as lignocellulosic materials. WB is one of the most important agricultural by-products from wheat milling industry and biorefineries. Annually, over 150 million tons of WB are produced in the world which are basically used for animal feed (Prückler et al., 2014). WB is a complex matrix of cellulose (40–50% of dry matter), hemicellulose (25–35% of dry matter), and lignin (15–20% of dry matter), it constitutes a valuable resource to produce platform molecules and bioethanol (Celiktas et al., 2014; Prückler et al., 2014). Here, we investigated the pretreatments of WB and DWB using RTIL [C2mim][OAc] pure or diluted in aqueous solution. The influence of main operating conditions such as pretreatment temperature, time, RTIL percentage (RTIL to water ratio), and biomass loading (RTIL to WB or DWB ratio) were investigated and optimized through of PLS Methodology never described in the literature for biomass pretreatment. This modeling technique establishes the relationship between a set of predictors (or factors in an Experimental Design) and response variables which can be used to investigate the optimum process conditions (Yañez et al., 2012). The obtained model was experimentally validated and supported by a comprehensive study including chemical composition analysis and structural characterization.

## Materials and Methods

### Wheat Bran, Chemicals, and Enzyme

Wheat Bran (WB) was provided by Chamtor, Bazancourt, France. For the preparation of DWB, WB was washed with water at 40°C during 10 min (solid/liquid ratio: 1/10) and filtrate through a glass filter (40–100 μm pososity). Four successive washings were performed to obtain a total destarching. [C2mim][OAc] were purchased from Solvionic S.A. (Verniole, France). Standards (arabinose, galactose, glucose, xylose, cellobiose, and cellotriose) were purchased from Sigma Aldrich (Steinheim, Germany). All other chemical reagents were purchased from commercial sources in France and used as received. Cellic CTec2 enzymatic cocktail including both cellulolytic and hemicellulolytic activities was supplied by Novozymes (Bagsvaerd, Denmark) and prepared at 15 FPU/g of WB or DWB for the production of hydrolysates rich in glucose and xylose. The value of 2.0 mg of reducing sugar as glucose from 50 mg of filter paper (4% conversion) in 60 min has been designated as the intercept for calculating filter paper cellulase units (FPU) by IUPAC.

### Pretreatment of WB and DWB Samples With RTIL-Water Mixture

Before the pretreatment, WB and DWB samples were dried at 105°C during 24 h. The dried samples were then weighted in a screw capped Teflon tube and 10 mL of pure RTIL or RTIL-water mixture were added. The pretreatment was performed in a silicone oil bath under vigorous stirring (700 rpm) at various temperature and reaction time ranges as given in Table 1. After incubation, the pretreated sample was cooled and mixed with 20 mL of anti-solvent (ultrapure water, 20°C) for 30 min. The resulting suspension was subsequently centrifuged (10.1733 g) (Allegra® 64R Beckman Coulter, Indianapolis, United States) at 20°C for 20 min. This step was repeated 6 times until amber-colored suspension became clear with conductivity lower than 200 μS/cm. This extensive washing step ensured minimization of residual IL to prevent interference with the enzymatic hydrolysis (Liang et al., in press). The resulting insoluble substrate was then freeze-dried at room temperature for 24 h and collected for subsequent enzymatic hydrolysis. Recovered permeate solutions (solvent-RTIL mixture) were stored for a subsequent RTIL-recycling.

**Table 1:** Experimental range of levels and coding of factors used to evaluate the sugar yields after pretreatment by [C2mim][OAc].

**Factors**	**Coding**	**Range of levels**		
		**Low**	**Middle**	**High**
Temperature (°C)	X_1_	25	87.5	150
Time (min)	X_2_	40	110	180
RTIL percentage (% v/v)	X_3_	10	55	100
Biomass loading (% w/v)	X_4_	2.0	3.5	5.0

### Enzymatic Hydrolysis

The enzymatic hydrolysis procedure is adapted from Auxenfans et al. (2014b, 2017a). The Cellic CTec2-catalyzed hydrolysis of the different WB and DWB substrates (untreated or pretreated) was carried out in 1 mL Eppendorf tubes. For hydrolysis reaction, 20 mg of sample was added to 590 μL of citrate-phosphate buffer (50 mM, pH 5.5), 100 μL of fucose preparation (2 mg/mL) which was used as internal standard and 310 μL of Cellic CTec2 preparation (0.974 FPU/mL) with the aim to have an enzyme loading of 15 FPU/g of WB or DWB. The mixture was incubated in a thermomixer (700 rpm) (Eppendorf Thermomixer C, Eppendorf, Hambourg, Germany) at 50°C for 72 h. The reaction was stopped by incubating the mixture at 90°C for 20 min. Then, the sample was dilute (100x) in ultrapure water and filtered (0.22 μm Syringe PTFE filter) prior to quantify the sugar content by High Performance Anion Exchange Chromatography (HPAEC-PAD) (Dionex ICS 5000+ DC, Thermo Fisher Scientific, Massachusetts, United States). The hydrolysis reaction was repeated in duplicate.

### Sugar Analysis

To determine the sugar concentrations a method was developed by HPAEC using an analytical CarboPac PA-20 (150 ×3 mm) column equipped with a guard column (30 ×3 mm) (DIONEX, Thermo Fisher Scientific, Massachusetts, United States) and kept at 25°C. Elution was carried out at a flow rate of 0.5 mL/min. A gradient method was used for the separation of monosaccharides and disaccharides using solvent A: 10 mM NaOH, solvent B: 200 mM NaOH, and solvent C: ultra-pure water. The gradient consisted of 25% A and 75% C (0–20 min) followed by a linear increase of A to 100% (20–25 min) then a linear decrease of A to 0% and a linear increase of B to 27.5% and D to 62.5% (25–35 min) after the gradient was maintained to 27.5% B and 62.5% D (35–50 min) followed by a linear increase of B to 100% and a linear decrease of D to 0% (50–60 min). The column was then regenerated with 100% B (60–80 min) and equilibrated with 25% A and 75% C (20 min). The injection volume was 10 μL. Quantification was based on calibration curve using standard sugar solutions (0–0.1 g/L). The retention times of arabinose, galactose, glucose, xylose, mannose, cellobiose, and cellotriose were: 7.90, 9.90, 11.50, 13.6, 14.60, 34.8, and 45.5 min, respectively. The sugar concentrations were expressed in (g/L) from total of each sugar contained in native biomass.

For statistical analysis, the relative glucose and xylose yields for both untreated and pretreated samples were determined after 72 h of enzymatic hydrolysis according to the following equations:

(1) $$\begin{array}{l} \;Glucose\;yield\;\left(Y_{1}\right)\left(\%\right)\\ =\frac{glucose\;released\;after\;enzymatic\;hydrolysis\;\left(g\right)\;}{glucose\;of\;untreated\;biomass\;\left(g\right)} \end{array}$$

(2) $$\begin{array}{l} \;Xylose\;yield\;\left(Y_{2}\right)\left(\%\right)\\ =\frac{xylose\;released\;after\;enzymatic\;hydrolysis\;\left(g\right)}{xylose\;of\;untreated\;biomass\;\left(g\right)} \end{array}$$

### Experimental Design and Statistical Analysis

In this research, two kinds of models were performed (a linear and a second order models) to estimate the effect of four factors pertaining ionic liquid pretreatment on the response. Thus, pretreatment temperature (X1), time (X2), RTIL percentage i.e., RTIL to water ratio (X3) and biomass loading (X4) were studied on glucose (Y1) or xylose (Y2) yields, the two major monosaccharides released by enzymatic hydrolysis from WB and DWB. The highest and lowest levels of factors were selected based on the results achieved in preliminary tests. The levels of factors are shown in Table 1. The experiments were designed by Statgraphics Centurion XVIII® (FranceSTAT, Neuilly sur Seine, France) and MODDE 12® (Umetrics, Umea, Sweeden) software. The experimental design consists of 2^4^ factorial points and 2 central points. A total of 18 experiments were randomly performed in duplicate with 2 repetitions of central point (Table 2).

**Table 2:** Experimental design matrix of four factors and data for glucose and xylose yields after pretreatment of WB and DWB with [C2mim][OAc].

**Pretreatment**	**Temperature (°C) X_**1**_**	**Time (min)X_**2**_**	**RTIL (% v/v) X_**3**_**	**Biomass loading (% w/v)X_**4**_**	**WB**		**DWB**	
					**Glucose yields^a^ Y_**1**_**	**Xylose yields^b^Y_**2**_**	**Glucose yields Y_**1**_**	**Xylose yieldsY_**2**_**
Untreated	n.a	n.a	n.a	n.a	0.10 ± 0.00^c^	0.28 ± 0.02	0.26 ± 0.01	0.22 ± 0.01
P1	150	40	10	2.0	0.30 ± 0.00	0.45 ± 0.00	1.00 ± 0.09	0.79 ± 0.04
P2	25	180	10	2.0	0.17 ± 0.00	0.25 ± 0.00	0.16 ± 0.03	0.18 ± 0.00
P3	25	40	100	2.0	0.15 ± 0.00	0.36 ± 0.01	0.14 ± 0.02	0.16 ± 0.03
P4	150	180	100	2.0	0.26 ± 0.01	0.07 ± 0.08	0.80 ± 0.01	0.10 ± 0.03
P5	25	40	10	5.0	0.50 ± 0.16	0.74 ± 0.17	0.73 ± 0.03	0.58 ± 0.03
P6	150	180	10	5.0	0.27 ± 0.16	0.39 ± 0.05	0.97 ± 0.04	0.82 ± 0.01
P7	150	40	100	5.0	0.27 ± 0.03	0.54 ± 0.06	0.75 ± 0.01	0.41 ± 0.02
P8	25	180	100	5.0	0.53 ± 0.02	0.69 ± 0.01	0.17 ± 0.01	0.17 ± 0.05
P9	25	40	10	2.0	0.10 ± 0.00	0.63 ± 0.03	0.22 ± 0.01	0.23 ± 0.00
P10	150	180	10	2.0	0.84 ± 0.03	0.54 ± 0.01	1.00 ± 0.08	0.58 ± 0.04
P11	150	40	100	2.0	0.48 ± 0.19	0.22 ± 0.00	0.93 ± 0.00	0.29 ± 0.00
P12	25	180	100	2.0	0.76 ± 0.02	0.76 ± 0.07	0.86 ± 0.07	0.64 ± 0.01
P13	150	40	10	5.0	0.87 ± 0.02	0.91 ± 0.03	1.00 ± 0.11	0.54 ± 0.05
P14	25	180	10	5.0	0.69 ± 0.02	0.84 ± 0.11	0.20 ± 0.06	0.17 ± 0.06
P15	25	40	100	5.0	0.12 ± 0.01	0.26 ± 0.19	0.37 ± 0.02	0.56 ± 0.02
P16	150	180	100	5.0	0.86 ± 0.18	0.51 ± 0.01	0.95 ± 0.02	0.53 ± 0.09
P17	87.5	110	55	3.5	0.26 ± 0.01^d^	0.39 ± 0.03^d^	0.17 ± 0.02^d^	0.40 ± 0.01^d^
P18	87.5	110	55	3.5	0.31 ± 0.01^d^	0.46 ± 0.16^d^	0.22 ± 0.00^d^	0.35 ± 0.00^d^

a *$Glucose\;yield\;\left(Y_{1}\right)=\frac{glucose\;released\;after\;enzymatic\;hydrolysis\;\left(g\right)}{glucose\;of\;untreated\;biomass\;\left(g\;\right)}$*.

b *$Xylose\;yield\;\left(Y_{2}\right)=\frac{xylose\;released\;after\;enzymatic\;hydrolysis\;\left(g\right)}{xylose\;of\;untreated\;biomass\left(g\;\right)}$*.

c *Mean ± SD of duplicate*.

d *Central points*.

*n.a, not applicable*.

The experimental design was submitted to goodness of fit routine using a first and second order equation by MODDE 12® software as shown in Equations (3) and (4).

(3) $$\begin{array}{l} Y_{1,2}=\beta_{0}+\overset{4}{\underset{i=1}{\sum }}\beta_{i}X_{i}+\overset{4}{\underset{i,j=1}{\sum }}\beta_{ij}{X_{i}X}_{j}+\;\varepsilon \end{array}$$

(4) $$\begin{array}{l} Y_{1,2}=\beta_{0}+\overset{4}{\underset{i=1}{\sum }}\beta_{i}X_{i}+\overset{4}{\underset{i=1}{\sum }}\beta_{ii}X_{i}^{2}+\overset{4}{\underset{i,j=1}{\sum }}\beta_{ij}{X_{i}X}_{j}+\;\varepsilon \end{array}$$

Where *Y*_1_ is the response variable for glucose and *Y*_2_ for xylose, X*i*'s are the independent factor variables. β_0_, β_*i*_, β_*ii*_, and β_*ij*_ are the constant, linear, quadratic and two factor interaction coefficients, respectively. Each coefficient in the second-order polynomial model was calculated and the possible interaction effects of pretreatment factors on the glucose and xylose yields were obtained. The experimental error ε is assumed to be randomly drawn from a normal distribution with a mean of 0 and a standard variance equal to σ^2^.

The method used to fit the models was PLS supported by NIPALS algorithm (Wold et al., 2001). This method has the properties of support collinearity, no-normality, missing data, and statistical noise (Ferrer, 2007). These conditions allow fitting phenomena with high variability improving the features of the standard method MLR than cannot explain these kinds of situations. We applied a Variable Importance to the Projection (VIP) as part of the PLS computes (Eriksson et al., 2013). This analysis creates a hierarchy of the explanatory capacity of the independent variables on the variable(s). The VIP is based on the weighted sum of squares of the weights of the model factors, allowing a hierarchical ordering of the independent variables (Wold et al., 2001).

The significance of the developed models was checked by Analysis of Variance (ANOVA). The quality of the goodness of fit of second-order model was expressed by the coefficient of determination *R*^2^, $R_{\text{adj}}^{2}$, and the statistical significance of model was determined by the *F*-test (*p* < 0.05). Optimization (maximum of glucose and xylose yields) was also determined by PLS method and D-Desirability Function. A Multiple Response Optimization (MRO) for Glucose and Xylose yields was also performed using Nelder-Mead Simplex Method (Eriksson et al., 2013). VIP charts and surface response plots were drawn using MODDE 12 software to illustrate the effects of pretreatment factors on sugar yields.

### Characterization of WB and DWB Samples

#### Compositional Analysis

The moisture content of samples (g water/100 g WB or DWB) was determined by drying the samples (1 g) in a hot air oven at 105°C overnight. Protein content was determined using the Kjeldahl method. A factor of 5.7 was used for conversion from nitrogen (N) to protein content (AOAC Official Method, 2001). In order to obtain the destarched WB sample, the soluble starch was removed by washing the WB in hot water (50°C). Starch content in WB and in DWB was quantified with the kit K-TSHK from Megazyme (Megazyme, Pontcharra Sur Turdine, France). Relative contents of cellulose, hemicellulose, lignin and ash were determined according to the Goering and Van Soest method (Goering and Van Soest, 1970). Before the experiment, solid samples were dried at 105°C overnight and all the measurements were conducted duplicate. Five hundred milligram of sample (W0) were mixed with 100 mL of neutral detergent at 100°C for 1 h. After filtration and washing with water and acetone, the residue was dried at 105°C for 8 h and then weighed (W1). The residue was then mixed with 100 mL of acid detergent, 0.5 g of sulfite de sodium and 2 mL de decahydronaphtalene at 100°C for 1 h to remove hemicellulose. After this step, the residue was dried at 105°C for 8 h and weighed (W2). Then, the residue was treated with 72% H_2_SO_4_ at 25°C for 3 h to remove cellulose. After the removal of cellulose, the residue was dried at 105°C for 8 h and weighed (W3). Finally, the residue was incinerated at 550°C for 3 h to remove lignin. After incineration, the residue was dried and weighed (W4). The contents of cellulose, hemicellulose, lignin, and ash were calculated as follows:

(5) $$\begin{array}{l} Hemicellulose\;\left(\%\right)=\frac{\left(W_{1}-W_{2}\right)}{W_{0}}\;\times \;100 \end{array}$$

(6) $$\begin{array}{l} Cellulose\;\left(\%\right)=\frac{\left(W_{2}-W_{3}\right)}{W_{0}}\;\times \;100 \end{array}$$

(7) $$\begin{array}{l} Lignin\;\left(\%\right)=\frac{\left(W_{3}-W_{4}\right)}{W_{0}}\;\times \;100 \end{array}$$

(8) $$\begin{array}{l} Ash\;\left(\%\right)=\frac{W_{4}}{W_{0}}\;\times \;100\; \end{array}$$

#### Structural and Morphological Characterization of WB and DWB Samples

The morphology of untreated/pretreated WB and DWB samples was investigated by Scanning Electron Microscopy (SEM). In the case of cellulose samples, the gold metallization prior to SEM analysis could induce physical changes and possible artifacts due to the action of the vacuum and/or the gold deposit heating (Husson et al., 2011). Thus, another classical method was preferred for LCB visualization, which consisted of observation in low-vacuum mode (under partial vacuum pressure of water) without any sample preparation step. The microscope was an environmental high-resolution electron scanning microscope QUANTA 200 FEG (FEI Company, USA) with a LF (Large Field) detector. The conditions of observation were the following: acceleration voltage between of 2 kV, work distance between 5 and 9 mm and pressure between 0.5 and 2 m bar. ^13^C NMR spectra of untreated and pretreated samples were acquired at 25°C by using Cross-Polarization Magic Angle Spinning Nuclear Magnetic Resonance spectroscopy (CP-MAS ^13^C NMR) on a Bruker DRX-500 spectrometer equipped with a 4 mm probe operating at 125.7452 MHz (^13^C channel). Samples were spun with a MAS speed of 5 kHz. Calibration of ^13^C spectra was externally performed using ethylbenzene as a reference. The NMR spectra were processed using Brucker's Topspin 3.1 windows processing software.

## Results

The temperature and duration time of IL-pretreatment are important parameters for an efficient LCB deconstruction. Indeed, we have shown, in previous work, a significant effect on improving enzymatic saccharification after IL pretreatment on cellulose or woody residues from 25°C for 20 min or 45°C for 40 min, respectively (Auxenfans et al., 2012, 2014a). However, an increase in the temperature or duration of IL-pretreatment allow to achieve higher sugar yields usually implemented at temperature between 80°C and 160°C from hours to overnight regardless of biomass (Olivier-Bourbigou et al., 2010; Brandt et al., 2013; Badgujar and Bhanage, 2015; Liang et al., in press). Another point that can be connected is the biomass loading, especially with pretreatment with pure IL due to high viscosity (Alayoubi et al., 2019). These studies led us to the choice of experimental range gathered in Table 1. The focus of the present work was to optimize pretreatment conditions of IL-water mixture allowing on one hand to reduce viscosity, and thus facilitating the handling in the process, and, on the other hand, to reduce cost due to IL.

### Chemical Composition and RTIL-Water Mixture Pretreatment of WB and DWB

As starch can be reserved for other uses and also its content can affect the enzymatic performances on cellulose and hemicellulose, another study was conducted in parallel on DWB. After washing WB in hot water, the starch percentage in the samples was decreased by a factor of 3.7 times (Table 3). LCB polysaccharides in raw materials account for 48.4–65.6% of the dry matter where 14.1–21.1% correspond to the cellulose content and 34.2–40.9% to the hemicellulose fraction. Total lignin content of all samples varied between 4.7 and 12.6% and small amounts of various compounds (0.5% ash content) were also found in WB and DWB samples (Table 4). The relative composition of remains almost unchanged after the pretreatment.

**Table 3:** Relative chemical composition of raw WB and DWB.

	**WB (%)**	**DWB (%)**
Moisture^a^	9.05 ± 0.16^e^	5.41 ± 0.13
Starch^b^	24.50 ± 0.00	6.60 ± 0.00
Protein^c^	16.42 ± 0.48	16.33 ± 0.78
Arabinose^d^	11.43 ± 1.18	12.41 ± 0.36
Galactose^d^	0.83 ± 0.08	0.81 ± 0.09
Glucose^d^	25.69 ± 2.87	13.57 ± 0.07
Xylose^d^	16.61 ± 0.89	19.04 ± 0.47

a *Determined at 105°C during 24 h*.

b *Determined after washing in hot water*.

c *% N × 5.7*.

d *Determined by HPAEC-PAD after H_2_SO_4_ hydrolysis*.

e *Mean ± SD of duplicate*.

**Table 4:** Relative composition of polymers and ash of WB and DWB before and after pretreatment with [C2mim][OAc]^a^.

**Substrate**	**Cellulose**	**Hemicellulose**	**Lignin**	**Ash**
WB-untreated	14.13 ± 6.91^b^	34.24 ± 1.77	4.72 ± 0.47	0.54 ± 0.49
WB-[C2mim][OAc]-water mixture^c^	15.43 ± 3.15	36.28 ± 7.35	6.44 ± 0.49	0.25 ± 0.06
DWB-untreated	21.13 ± 3.25	40.85 ± 4.50	12.64 ± 4.10	0.46 ± 0.13
DWB-[C2mim][OAc]-water mixture^d^	23.35 ± 1.42	42.27 ± 4.01	10.78 ± 1.81	0.15 ± 0.05

a *Relative Chemical Composition (g/ 100 g of dry matter) determined by Goering and Van Soest (1970) method*.

b *Mean ± SD of duplicate*.

c *Pretreatment leading to higher sugar yields [150°C, 40 min, RTIL 10% v/v, biomass loading 5% (w/v)]*.

d *Pretreatment leading to higher sugar yields [150°C, 40 min, RTIL 10% v/v, biomass loading 5% (w/v)]*.

**Figure 1:**
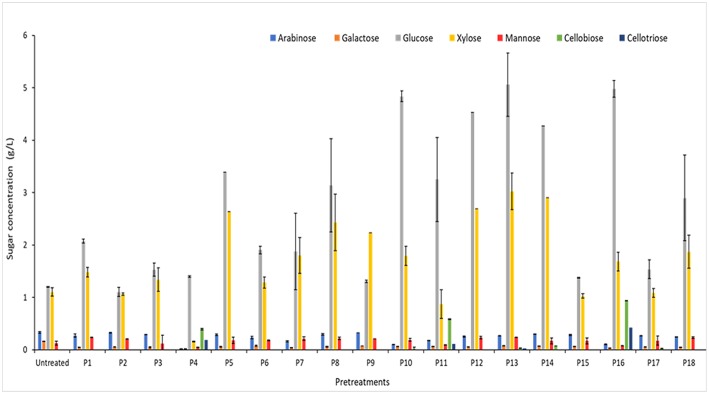
Sugar composition of WB after 72 h of hydrolysis with Cellic CTec2 at 15 FPU/g for different pretreatments. Hydrolysis were performed in duplicate with 2% w/v of WB.

**Figure 2:**
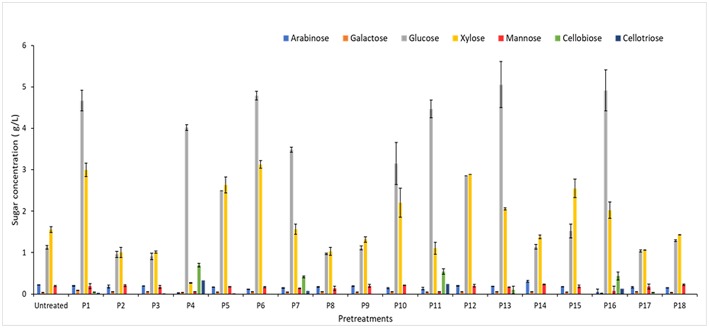
Sugar composition of DWB after 72 h of hydrolysis with Cellic CTec2 at 15 FPU/g for different pretreatments. Hydrolysis were performed in duplicate with 2% w/v of DWB.

The influence of pretreatment by [C2mim][OAc]-water mixtures on saccharification performances of WB and DWB samples was then investigated through the sugar yields obtained after 72 h of hydrolysis with the cocktail enzymatic Cellic CTec2. Untreated WB and DWB samples were also hydrolyzed with Cellic CTec2 under the same reaction conditions. Beyond the potential interest in enzymatic hydrolysis, this reaction can be used as test to assess the efficiency of a pretreatment in disrupting the LCB. The pretreatments were performed at different levels of temperature (25–100°C), time (40–180 min), percentage of [C2mim][OAc] (10–100% v/v) and biomass loading (2–5% w/v) following the experimental design shown in Table 2. The sugar production obtained after 72 h of enzymatic hydrolysis for untreated and pretreated WB and DWB were plotted as a function of pretreatments (Figures 1, 2). As seen on the figures, the two major monosaccharides released after the enzymatic hydrolysis were glucose and xylose in agreement with the initial composition.

### Model Fitting

Next, the effect of pretreatment factors was further investigate and optimize on the two main sugar released after the enzymatic hydrolysis through a designed experiment using PLS methodology. Thus, Table 2 shows the design matrix generated by PLS statistical tool according to Equations (1) and (2) as well as the xylose and glucose yields obtained after 72 h of enzymatic hydrolysis of WB and DWB. Pretreatment with [C2mim][OAc]-water mixture increased significantly the glucose and xylose yields of about 3–10 folds as compared to those of untreated samples. A second-order polynomial model was then used to fit the response as shown earlier in Equation (4). Thus, the coefficients β_0_, β_*i*_, β_*ii*_, and β_*ij*_ of each model estimated by the MODDE 12^®^ software were obtained which are shown in Table 5.

**Table 5:** Regression coefficients and ANOVA of quadratic model.

**Coefficients**	**WB**		**DWB**	
	**Glucose Yield Y_**1**_**	**Xylose YieldY_**2**_**	**Glucose Yield Y_**1**_**	**Xylose YieldY_**2**_**
Constant β_0_	0.2809	0.4199	0.1952	0.3634
**Linear**				
β_1_	−0.0223	−0.0659	0.2789	0.0678
β_2_	−0.0002	−0.0452	−0.0564	−0.0413
β_3_	0.0191	−0.0117	−0.0515	0.0056
β_4_	0.0901	0.1086	0.0377	0.0636
**Quadratic**				
β_11_	0.0129	0.0104	0.1652	0.0475
β_22_	0.0225	−0.0252	0.0334	−0.0189
β_33_	0.1499	0.0177	0.0643	−0.0009
β_44_	−0.0204	0.0105	0.0726	−0.0236
**Interaction**				
β_12_	−0.1465	−0.0792	0.0409	0.0565
β_13_	0.0000	0.0001	0.0007	0.0103
β_14_	−0.0017	0.0874	−0.0456	−0.0185
β_23_	0.0908	0.1324	0.0493	0.0089
β_24_	0.0013	0.0193	−0.0471	−0.0661
β_34_	−0.1493	−0.0383	−0.0265	0.0025
Model *F*-value	35.28^*^	62.90^*^	54.25^*^	83.57^*^
*p*-value Prob > F	<0.0001	<0.0001	<0.0001	<0.0001
*R*^2^	0.96	0.97	0.97	0.98
$R_{\text{Ajusted}}^{2}$	0.93	0.96	0.95	0.97
RSD (%)	6.10	4.30	7.55	2.69

* *p < 0.001*.

**Figure 3:**
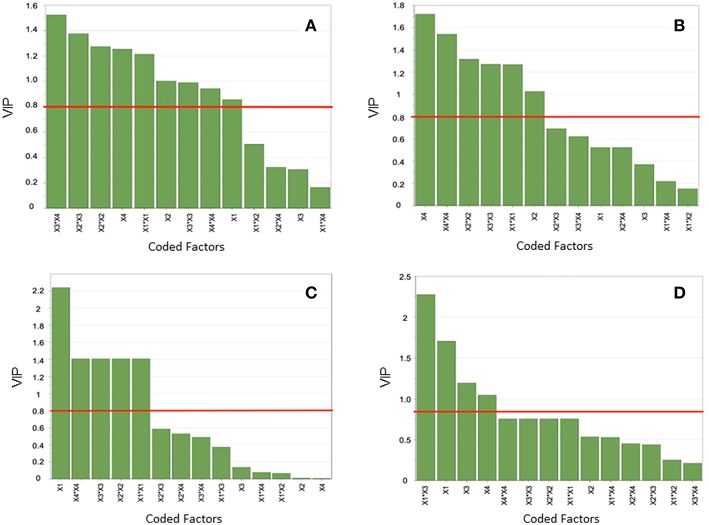
Effect of pretreatment factors by Variable Importance to Projection (VIP) charts on glucose and xylose yields: **(A,B)** for WB and **(C,D)** for DWB. Any factor (green bars) beyond the specified significance level of 0.8 (orange line) is statistically significant (*p* < 0.05).

**Figure 4:**
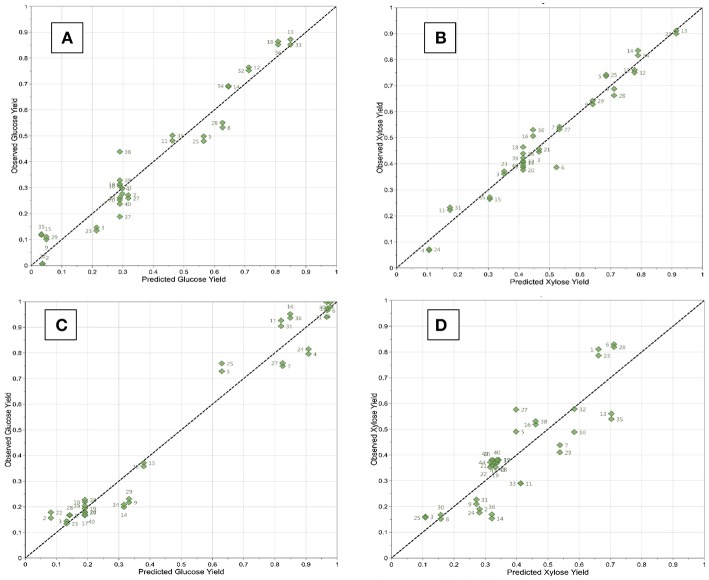
Correlation graphs between the predicted and observed experimental glucose and xylose yields values for WB **(A,B)** and DWB **(C,D)**, respectively.

Analysis of variance (ANOVA) was performed to test the significance of the developed model and VIP charts were utilized to evaluate the linear, quadratic and interaction effects on the response. ANOVA analysis revealed that model *F*-values ranged from 35.3 to 83.6 and the values of “Prob > F” were <0.001 (Table 5). The calculated determination coefficients (*R*^2^) were in agreement with the adjusted determination coefficients (*R*_2_Adj). The Relative Standard Deviation (RSD%) of models were lower than 10% (Table 5). As part of PLS analysis, VIP values were graphically represented as a function of the coded factors: temperature (X_1_), time (X_2_), RTIL percentage (X_3_), and biomass loading (X_4_) (Figure 3). Thus, on the Figure 3, any factor (Bar) beyond the specified level of 0.8 (orange horizontal line) was considered as statistically significant (*p* < 0.05). Furthermore, the experimental data and the predicted values were closely grouped close to the line of best fit as shown in Figure 4.

**Figure 5:**
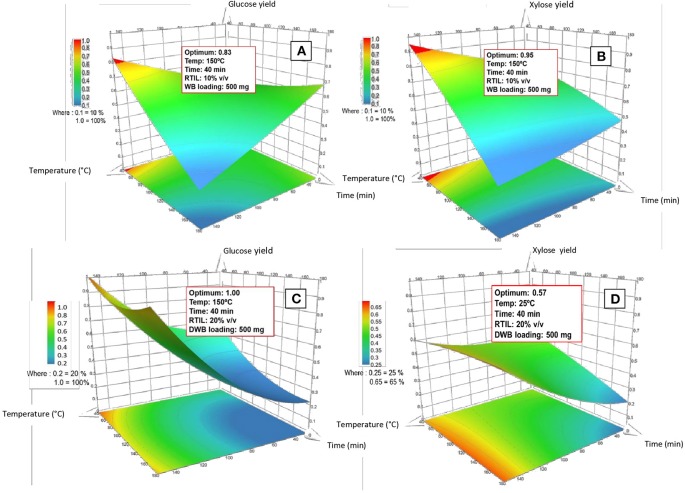
Response surface plots of sugar release after enzymatic hydrolysis for WB **(A,B)** and DWB **(C,D)** as a function of time (40–180 min) and temperature (25–150°C).

Based on the obtained quadratic models, response surface (RS) plots were drawn to evaluate the effect (simple, quadratic, and interaction) of the different factors within the range studied in the present work. The effect on glucose yields are shown in Figures 5A,C for WB and DWB, respectively and the effect on xylose yields in Figures 5B,D for WB and DWB, respectively. Except for DWB xylose yield, the trend showed a better sugar yield with an increase in the temperature at low pretreatment times.

### Morphology and Structure of WB and DWB

**Figure 6:**
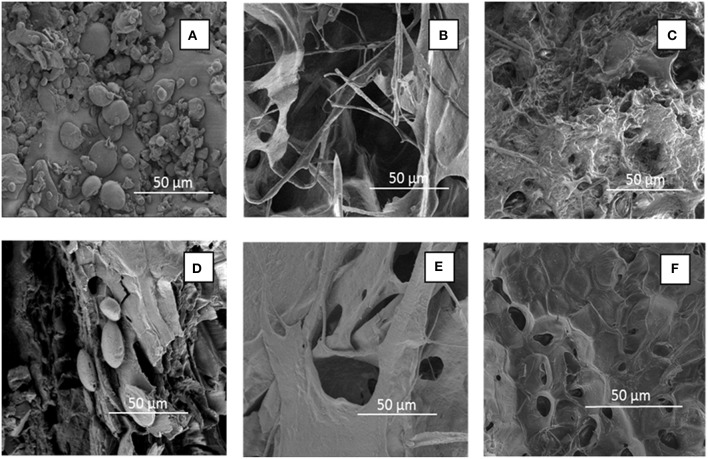
SEM images (x 500) of untreated WB **(A)** and DWB **(D)** and pretreated with pure [C2mim][OAc] WB **(B)** and DWB **(E)** and with [C2mim][OAc]-water mixtures (RTIL content of 10%) [WB **(C)** and DWB **(F)**] at 150°C for 40 min.

Finally, the morphological and structural changes on WB and DWB samples after pretreatment are illustrated in Figure 6 and Figure S1, respectively. The surface morphology of the WB and DWB samples before and after pretreatment was analyzed by SEM. As shown in Figure 6, the raw WB and DWB showed a surface compact, irregular and agglomerated as “flakelike” structures (Figures 6A,D) where bran starch granules can be randomly observed even after the destarching step (Figure 6D). Pretreatment with pure [C2mim][OAc] induced drastic morphological changes (Figures 6B,E). As compared to untreated samples, large cavities appeared and the matter seemed completely disorganized. After RTIL-water mixture pretreatment at 150°C for 40 min of WB or DWB (Figures 6C,F), a more porous and expanded structure was observed as compared to untreated samples but less impacted than with pure IL pretreatment. In addition, solid state ^13^C NMR was used to characterize the structure of the WB and DWB samples before and after pretreatment with the aqueous [C2mim][OAc]. The ^13^C NMR (Figure S1) peaks were assigned according to data published in the literature (Ha et al., 1997; Gauthier et al., 2002; Locci et al., 2008). The region at 120–170 ppm and the peak at 56 ppm represent the signals assigned to the aromatic carbons and the aromatic the methyl ester groups of the lignin while the peaks shown at 170–178 ppm and 18–24 ppm represent the signal for the hemicellulose carboxyl and acetyl carbon groups, respectively (Sannigrahi et al., 2011). The intense signal occurring from 60 to 110 ppm represents the C-atoms of the cellulose in the samples. In particular, the signal representing the C4 of cellulose appeared from 83 to 92 ppm as broad signals. In particular, the signal centered at 83 ppm corresponds to the C4 of the amorphous cellulose while the shoulder at 89 ppm represents the C4 of the crystalline cellulose.

## Discussion

### Impact of RTIL-Water Mixture Pretreatment of WB and DWB on Enzymatic Hydrolysis

Pretreatment with diluted [C2mim][OAc] did not induce a real fractioning of constitutive cellulose/hemicellulose/lignin of WB whatever the presence or absence of starch (DWB) as the relative composition remains almost unchanged after the pretreatment (Table 4 and Figure S1). This behavior was in agreement with the results published in previous works where pure RTIL was used to pretreat the LCB (Auxenfans et al., 2014b; Husson et al., 2018). This suggests that the presence of water in the [C2mim][OAc] medium did not affected the specificitiy of RTIL mild pretreatment. Indeed, fractioning and partial degradation of polysaccharidic and polyphenolic polymers were minimized contrary to the chemical pretreatment such as hydrothermal or diluted-acid pretreatments (Chandra et al., 2007; Hendriks and Zeeman, 2009; Reisinger et al., 2013; Akhtar et al., 2016). The statements based on the comparison of chemical analyses before and after pretreatment with RTIL-water mixture were also supported by NMR spectroscopic measurements as illustrated in Figure S1. Indeed, no major modification was evidenced after the pretreatment for each biomass as specific peaks of hemicelluloses and lignins were still observed. It could be due either to the use of IL in water mixture or more specifically to the studied biomass. In effect, it has already been reported that pretreatment with pure [C2mim][OAc] induced partial removal or dissolution of lignin and extractives or induced deacetylation of hemicellulose of wood residues (Çetinkol et al., 2010; Auxenfans et al., 2014b). However, there is a consensus on the effect of IL on the lignin-carbohydrate complex disruption whatever the extent of fractionation and structural changes on cellulose (Singh et al., 2009; Husson et al., 2011). In addition, NMR spectra did not show characteristic peaks of IL in the pretreated WB or DWB, it can be thought that there is no or negligible amount of residual IL that may be entrapped in the pretreated biomass.

Whatever the pretreatment conditions (Figures 1, 2), the enzymatic hydrolysates are mainly composed of glucose and xylose and only small amounts of other sugars (arabinose, galactose and mannose) which were in agreement with the sugar composition (Table 3). This is consistent with the high cellulase (97.3 FPU/mL) and xylanase (12082 UI/mL) activities in the Cellic CTec2 cocktail. On the other hand, the low arabinosidase activity in the Cellic CTec2 cocktail (3.4 IU/mL for arabinosidase) did not allow for a significant release of arabinose in any case.

High yields of glucose or xylose can be achieved by enzymatic hydrolysis after RTIL-water pretreatment in mild conditions allowing to consider the reducing use of IL pretreatment to overcome the lignocellulosic recalcitrance prior to enzymatic production of C6 and C5 sugars.

### Model Fitting and Statistical Analysis

Utilization of RTIL for WB pretreatment in order to get maximum sugar yields after enzymatic hydrolysis might result in an expensive method to pretreat this biomass. Optimization of pretreatment factors is thus essential to obtain the most suitable levels of the process conditions, especially, the percentage of RTIL. The experimental data of glucose and xylose yields presented in Table 2 were used to fit the model (Equation 4) to predict the responses and identify the regression coefficients of the second order equation (Table 5). Based on coefficients presented in Table 5, the relation between the investigated factors and the glucose or xylose yields can be obtained.

ANOVA was implemented to test the significance of the established models. A model is considered significant if its “Prob > F” value (*p*-value) is <0.05 and its *F*-value is relatively high (*F* > 4) which implies that there is only a 0.05% chance that a “Model *F*-value” could occur due to noise. In this study, the model *F* values were ≥35.3 indicating that all models were highly significant at the 95% confidence level (Table 5). Furthermore, the *p* value was *p* < 0.0001 which indicated that the quadratic model explains adequately the linear, quadratic and interaction effects on the response, in other words, the regression equation (Equation 4) was adequate to explain the glucose and xylose yields. According to the ANOVA results, the lack of fit was not significant for all response variables (*p* > 0.05) which confirm the goodness of the model. The fitness of the quadratic model was checked by means of the coefficients of determination. As can be seen from Table 5, the coefficients of determination (*R*^2^) ranged from 0.960 to 0.980 for both glucose and xylose yields implying a 96–98% variability of response explained. That is, only about 2–4% of the total variance could not be explained by the model. In general, quadratic model having *R*^2^ values higher than 0.90 are considered as models with a high degree of correlation (Cadoche and López, 1989; Myers et al., 2016). Moreover, the coefficient of adjusted determination (*R*_2Adj_) was calculated as an accurate measure of regression model quality. Thus, the high *R*_2Adj_ values (0.93–0.97) implied a high significance of the model. Finally, the precision and model reproducibility were evaluated by the RSD% that is defined as a percentage of error and whose value should not be higher than 10%. Consequently, according to the values of RSD (<10%) shown in Table 5 we can conclude that the current models for glucose and xylose yields are precise and reproducible.

To complete the PLS analysis, the effect of pretreatment factors on the response (sugar yields) was shown by means of VIP charts (Figure 3). It should be noticed that the weight of factors are different since 4 different and independent models (WB and DWB) were fitted for glucose and xylose yields obtained after enzymatic hydrolysis. From those models MRO was developed to obtain the maximum glucose and xylose yields for WB and in the same way another MRO for DWB. In the case of glucose yield for WB, the factors with the largest linear effects were the biomass loading (X_4_) followed by the time (X_2_) and the temperature (X_1_) while the RTIL percentage (X_3_) was not a significant factor (Figure 3A). For DWB, temperature was the most contributing factor affecting the glucose yield (Figure 3C). For both WB and DWB, the quadratic terms of temperature (X_1_X_1_), time (X_2_X_2_), RTIL percentage (X_3_X_3_), and biomass loading (X_4_X_4_) exerted a substantial effect on glucose yield at the 95% confidence level (Figures 3A,C). In particular, for WB, some interactions such as RTIL percentage and biomass loading (X_3_X_4_), time and RTIL percentage (X_2_X_3_) were, according to the model, statistically significant (*p* < 0.05). Otherwise, no interaction effects were observed (*p* > 0.005) in the case of DWB. In addition, for xylose yields, the linear effect of time (X_2_) and biomass loading (X_4_) and their quadratic terms had the most significant effect during the WB pretreatment (Figure 3B). In the case of DWB, it was affected by the single effect of temperature (X_1_), RTIL percentage (X_3_), biomass loading (X_4_) and the interaction of temperature, and RTIL percentage (X_1_X_3_) (Figure 3D).

Finally, the relationship between the observed values of glucose (Y_1_) and xylose (Y_2_) yields and the predicted values of the response from the models was shown in Figure 4. As illustrated in the Figures 4A–D, the values were closely grouped along the line of best fit which suggest a high degree of correlation between predicted and observed ones.

The PLS regression method to design of experiment utilize a type of validation named “Full Cross-Validation.” This PLS regression variant differs lightly from the routine fit used in other PLS applications, such as the Multivariate Calibration where a “trainee” data and a second “validation” set are normally used (Pontes et al., 2006; Cen et al., 2007). In resume, these statistical results corroborated the efficiency and reliability of the present regression models and demonstrated a good correlation between process factors and their effects on the response.

### Effect of Pretreatment Factors on Glucose and Xylose Yields and Optimums

As previously mentioned for WB, the VIP charts revealed that all factors present a clear quadratic tendency indicating that this effect was very critical for the model. Hence, the second order model was the most suitable to adjust the experimental data and to predict the glucose and xylose yields. These findings were supported by the curvatures and twists observed in the RS plots (Figures 5A,B). In fact, the RS plot for glucose yield showed a saddle curvature while for xylose yield the RS plot showed a clear convex curvature which is typical of a quadratic–shaped surface (Myers et al., 2016). Moreover, through PLS analysis made, the optimal conditions of WB pretreatment were determined, i.e.,: temperature (X_1_) = 150°C, time (X_2_) = 40 min, RTIL percentage (X_3_) = 10% (v/v) aqueous medium, and biomass loading (X_4_) = 5% (w/v) leading to yields predicted values of 83% in glucose and 95% in xylose (Figures 5A,B). Under the optimized conditions, slightly lower experimental yields were obtained: 77% for glucose and 90.4% for xylose. The residual values (that is: experimental value—model predicted value, expressed by absolute value) were estimated to be 6 and 4.6% for glucose and xylose yields, respectively.

As for WB, all of quadratic terms had a significant influence on glucose yield for DWB (Figure 3C). This phenomenon is reflected in the high yields obtained (100%) and graphically observed in the curvature of RS plot which present a steep slope (Figure 5C). In this context, if we compare the glucose yield of WB with that of DWB we can see clearly that in very similar experimental conditions, the performance of pretreatment would be significantly higher for DWB. The difference detected can be attributed to a higher level of RTIL (X_3_) (20% for DWB vs. 10% for WB). Maximum glucose yield (100%) was obtained under the following pretreatment conditions: temperature (X_1_) = 150°C, time (X_2_) = 40 min, RTIL percentage (X_3_) = 20% (v/v) in aqueous medium, and biomass loading (X_4_) = 5% (w/v) (Figure 5C).

On the contrary, in the case of xylose yield for DWB, the linear effects of X_1_, X_3_, and X_4_, the interaction effects of X_1_ and X_3_ were the ones who had the most significant influence in the fitted second order model (Figure 3D). This is agreement with the results obtained. Indeed, the RS plot (Figure 5D) presents a slight curvature concave and the optimum performance (57%) was very low when compared to that obtained for WB (95%). This may be partially explained by the effect not significant of interactions in the model (Figure 3D), in other words, the interactions have barely contributed to the xylose yield. In this context, a linear model could have been a more adequate solution to adjust the response. However, the slight curvature concave of the plot (Figure 5D) shows that the second order model was effectively the most appropriate to fit the xylose yields (lack of fit not significant). The optimal pretreatment conditions were: temperature (X_1_) = 25°C, time (X_2_) = 40 min, RTIL percentage (X_3_) = 20% (v/v) in aqueous medium, and biomass loading (X_4_) = 5% (w/v) (Figure 5C).

Under each optimal condition, yields obtained experimentally with DWB gave 92.1% for glucose and 53% for xylose, again slightly lower than the predicted ones, as for WB. Residual values were thus estimated at 7.9% for glucose and 4%n for xylose.

Finally, a MRO was performed by Nelder-Mead Simplex Method for each biomass (WB and DWB) in order to obtain best pretreatment conditions to maximize both glucose and xylose yields. The MRO for WB displayed optimums of glucose and xylose yields of 83.5 and 95.3%, respectively, which were obtained under the following conditions: temperature (X_1_) = 150°C, time (X_2_) = 40 min, RTIL percentage (X_3_) = 10% (v/v) in aqueous medium, and biomass loading (X_4_) = 5% (w/v). Furthermore, MRO for DWB revealed optimum of glucose and xylose yields of 100 and 56.1%, respectively. These optimum yields were obtained with a temperature (X_1_) = 150°C, time (X_2_) = 40 min, RTIL percentage (X_3_) = 20% of RTIL (v/v), and a biomass loading (X_4_) = 5% w/v. These yields are similar to those obtained by individual optimization with PLS approach, in other words, MRO maintain the yield values for both sugars. Based on the results mentioned above, we can observe that optimal pretreatment conditions for WB further improve the glucose yields while in the opposite case optimal pretreatment conditions for DWB improve the xylose yields. Moreover, pretreatment of WB and DWB with [C2mim][OAc]-water mixtures increased significantly the sugar yields of about 60–100% as compared to those of unpretreated biomass (10–20%). Results from this study were in agreement with others previous reports using [C2mim][OAc] and water as pretreatment media. Fu and Mazza (2011a) reported that a higher sugar yield (81%) was obtained after the [C2mim][OAc]-water pretreatment (50% of water content) of triticale straw at 150°C for 90 min. Perez-Pimienta et al. (2017) reported a glucan conversion efficiency of 98% for Agave bagasse biomass (40% water in [C2mim][OAc] media) and 83% for municipal solid waste biomass (50% water in [C2mim][OAc] media).

To sum up, optimal conditions to pretreat wheat bran by RTIL were determined. Our experimental design demonstrated the possibility to implement efficient pretreatment of wheat bran with diluted [C2mim][OAc] [10–20% (v/v)] in water within the range of study of the present work. Hence, this complete statistical study confirmed that the established models were appropriate to predict the sugar yields under different pretreatment conditions.

### Morphological and Textural Properties Associated With WB and DWB Pretreatment

To gain more insight into the effect of [C2mim][OAc]-water mixture, we focused then on the structural characterization of untreated and pretreated WB and DWB samples by performing SEM. For those analyses, we selected two pretreatment conditions leading to highest sugar yields. [C2mim][OAc]-water mixture pretreatments visibly induced a disorganization of WB and DWB leading to the formation of porous and expanded structure (Figures 6C,F) while more drastic effect was displayed with pure [C2mim][OAc] pretreatment (Figures 6B,E). The aqueous [C2mim][OAc] pretreatment contributed to the formation of a porous material that could account for increasing the enzymatic digestibility as already suggested by other authors (Singh et al., 2009; Hu et al., 2018). However, our optimized pretreatment conditions allowed preserving the structural integrity of cellulose as seen from the NMR signals of C4-carbon of amorphous and crystalline cellulose (Figure S1).

## Conclusion

This is the first work applying PLS methodology to optimize WB and DWB pretreatments with [C2mim][OAc]-water mixtures. Pretreatment conditions were optimized to achieve high glucose and xylose yields from WB biomasses by the proposed quadratic models. Highs glucose (83–100%) and xylose (57–95%) yields were obtained under optimized pretreatment conditions of temperature, time, concentration of [C2mim][OAc] in water and biomass loading. The complete statistical assessment confirmed that our established model is adequate and accurately predicts the glucose and xylose yield within the range of pretreatment conditions employed. Finally, the possibility to minimize the required RTIL amount to implement efficient pretreatment of WB and DWB was demonstrated which could have a great impact for further implementation at a large scale.

## Data Availability

All datasets generated for this study are included in the manuscript/Supplementary Files.

## Author Contributions

EH, CS, and CR conceived the project. EH, CS, MA-F, and JS conceived the experiments and wrote the manuscript. MA-F, DG, VL, IG, and HR achieved chemical composition, RTIL pretreatments, and enzymatic reactions experiments and analysis. MA-F and JS-T realized and interpreted the experimental design and statistical analysis. RR realized the SEM analysis. MA-F, EH, and CS coordinate the study and interpretations. All authors helped with drafting the manuscript.

### Conflict of Interest Statement

The authors declare that the research was conducted in the absence of any commercial or financial relationships that could be construed as a potential conflict of interest.
