# Supplementary material for: Wheat Bran Pretreatment by Room Temperature Ionic Liquid-Water Mixture: Optimization of Process Conditions by PLS-Surface Response Design
Source: Front Chem. 2019 Aug 23;7:585. doi: 10.3389/fchem.2019.00585 (PMC6716547; doi:10.3389/fchem.2019.00585)
Supplement: Supplementary file 1 [file Presentation_1.pptx]

## Slide 1
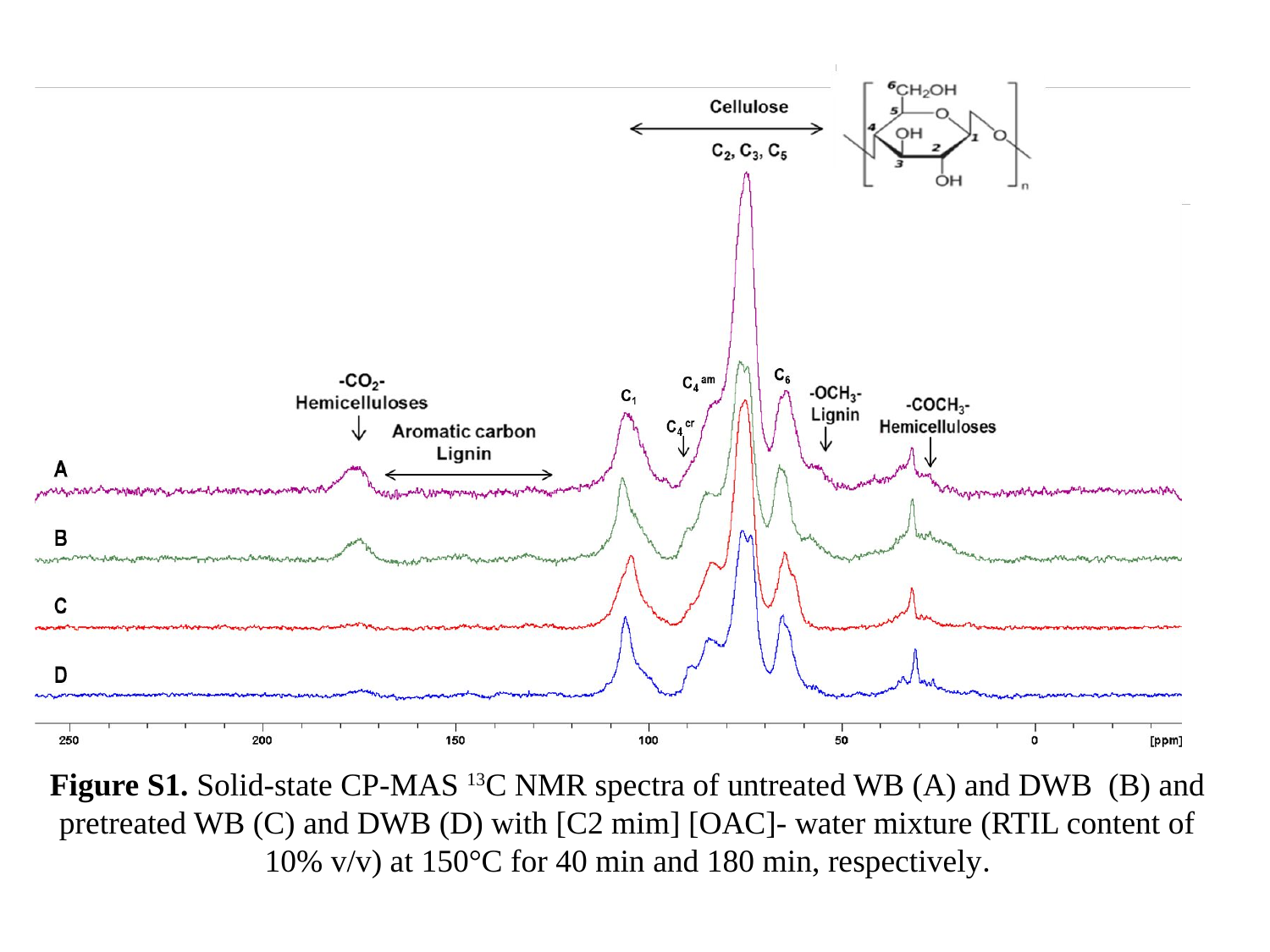

Figure S1. Solid-state CP-MAS 13C NMR spectra of untreated WB (A) and DWB (B) and pretreated WB (C) and DWB (D) with [C2 mim] [OAC]- water mixture (RTIL content of 10% v/v) at 150°C for 40 min and 180 min, respectively.
